# Supplementary material for: Acylcarnitines metabolism in depression: association with diagnostic status, depression severity and symptom profile in the NESDA cohort
Source: Transl Psychiatry. 2025 Feb 23;15:65. doi: 10.1038/s41398-025-03274-x (PMC11847943; doi:10.1038/s41398-025-03274-x)
Supplement: Supplementary file 1 — Supplemental materials [file 41398_2025_3274_MOESM1_ESM.docx]

***ONLINE SUPPLEMENT***

SUPLLEMENTARY INTRODUCTION 2

Figure 1. Acylcarnitine production and main roles in mitochondrial function. 2

SUPPLEMENTARY METHODS 3

Supplementary Figure 1 (Figure S1). Flow chart of subject’ inclusion. 3

Supplementary Table 1. Comparison of general characteristics of the main analytical sample and excluded subjects. 4

Metabolite profiling and data processing 5

SUPPLEMENTARY RESULTS 6

Supplementary Figure 2 (Figure S2). Distribution of metabolite levels. 6

Supplementary Figure 3 (Figure S3). Levels of correlation of ACs metabolites. 7

Supplementary Figure 4 (Figure S4). Levels of correlation of the three symptom scales: atypical energy-related (AES), anhedonic and melancholic. 8

Supplementary Figure 5 (Figure S5). Restricted cubic spline for C2 and the three symptom profiles. 9

Supplementary Table 2. Estimated means (and SE) of C2 levels after excluding participants with antidepressant use. 10

Supplementary Table 3. Association of C3 levels (divided in tertiles) with symptom profile. 11

Supplementary Table 4. Comparison of baseline characteristics of the subjects included at 6 year and those lost to follow-up from the main NESDA sample. 12

Supplementary Table 5. Association of metabolite levels with MDD severity and symptom profiles for pooled data. 13

Supplementary Table 6. STROBE Statement. 14

REFERENCES 16

**SUPLLEMENTARY INTRODUCTION**

**Figure 1. Acylcarnitine production and main roles in mitochondrial function.**

**Legend:** Abbreviations: CIT, cytosol; MIT, mitochondria; PEX, peroxisome; LC, long-chain; MC, medium-chain; SC, short-chain; CoA, coenzyme A; LACS, long-chain acyl-coenzyme A synthetase; CACT, carnitine/acylcarnitine translocase; CPT1 and CPT2, carnitine palmitoyl-transferase 1 and 2; CRAT, carnitine O-acetyltransferase; ATP, adenosine triphosphate; BCAA, branched-chain amino acid; OC Fatty Acid, Odd-chain fatty acid. Model based on Dambrova et al. (2022)^[3]^ and Li et al. (2019)^[4]^.

The main function of ACs is involved in long-chain fatty acids β-oxidation, where they are carriers transporting long-chain Fatty Acids into mitochondria after their activation by the link with coenzyme A via LACS. Long- and medium-chain acyl-CoAs are converted into LC Acylcarnines by CPT1 which is located on the outer mitochondrial membrane. Under catalysis of CACT, these LC Acylcarnines are imported through the mitochondrial membranes into the mitochondrial matrix (to be noted, part of the ACs involved are produced in peroxisomes). Then they are converted back to the corresponding long-/medium-chain acyl-CoAs by CPT2. These products will participate to β-oxidation for the production of ATP. The end products, acetyl-CoAs, are converted to acetylcarnitines (C2) by carnitine O-acetyltransferase (CRAT), which regulates the acetyl-CoA/free CoA ratio to prevent depletion of free CoA. Also, branched-chain amino acid (BCAA) and odd-chain fatty acids metabolization produce propionyl-carnitine (C3) which allows the creation of propionyl-CoA, substrate for the Krebs cycle that generate energy through the complete oxidation of acetyl groups derived from carbohydrates, fats, and proteins.

# **SUPPLEMENTARY METHODS**

## **Supplementary Figure 1 (Figure S1). Flow chart of subject’ inclusion.**

**
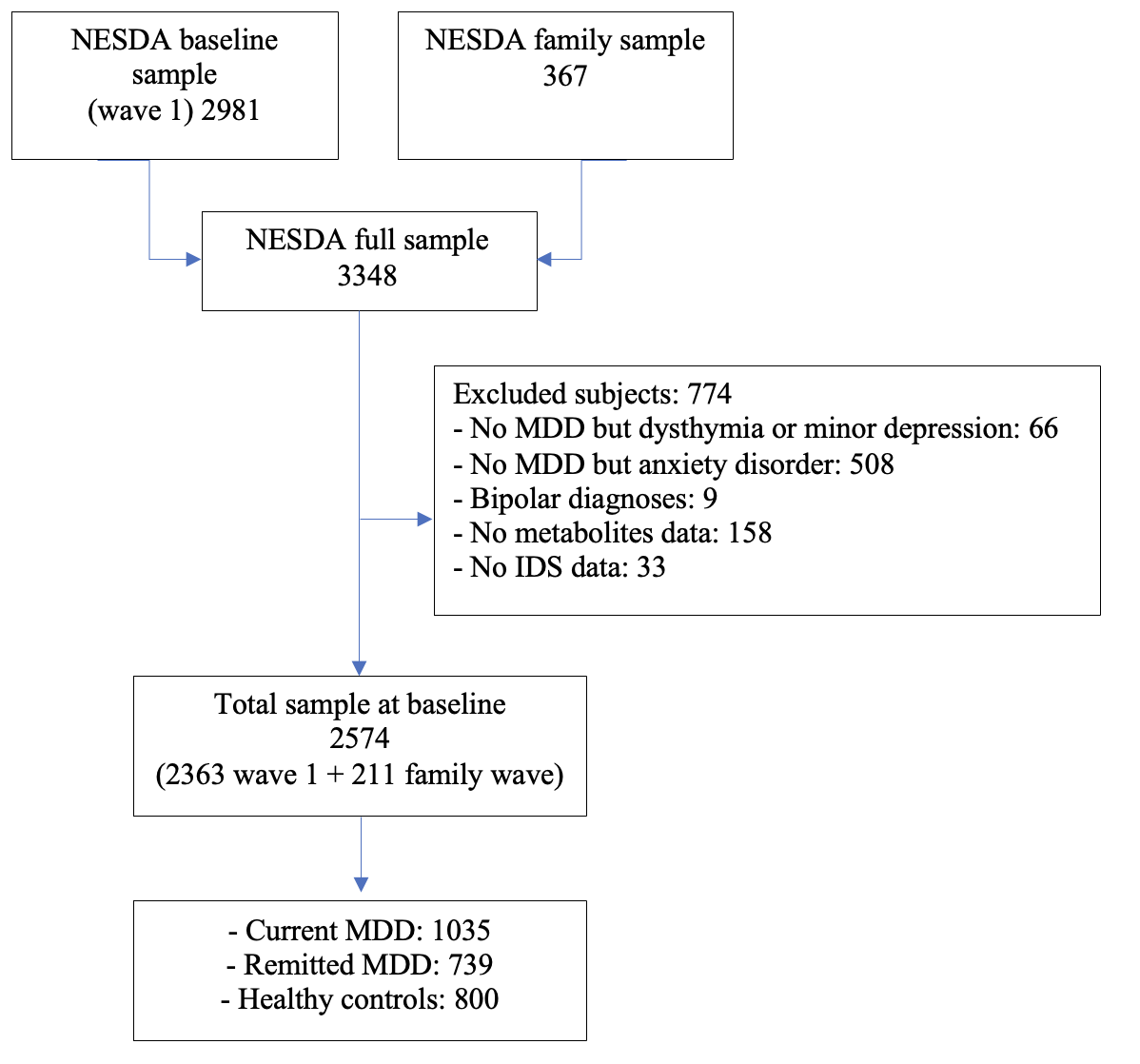
**

## **Supplementary Table 1. Comparison of general characteristics of the main analytical sample and excluded subjects.**

| **Table S1**: Comparison of general characteristics of the main analytical sample and excluded subjects. | | | | |
| --- | --- | --- | --- | --- |
|  | *Included (2440)* | *Excluded (509)* | *F / X2* | *P* |
| *Sociodemographic characteristics* | | | | |
| Age, years - mean±SD | 42.88±13.17 | 43.61±13.65 | 1.24 | 0.266 |
| Gender - n(%) |  |  | 0.003 | 0.953 |
| Male | 847 (34.7) | 176 (34.6) |  |  |
| Female | 1593 (65.3) | 333 (65.4) |  |  |
| Level of education, years - mean±SD | 12.36±3.29 | 12.14±3.39 | 1.69 | 0.195 |
| *Lifestyle and health* | | | | |
| Smoking status - n(%) |  |  | 0.94 | 0.332 |
| No smoker | 1559 (63.9) | 313 (61.6) |  |  |
| Current smoker | 881 (36.1) | 195 (38.4) |  |  |
| Physical activity - mean±SD |  |  |  |  |
| MET total | 3737.47  ±3696.26 | 3059.13 ±2837.28 | 0.09 | 0.769 |
| Chronic diseases - n(%) |  |  | 2.27 | 0.321 |
| None | 1106 (45.3) | 221 (43.4) |  |  |
| One disease | 777 (31.8) | 156 (30.6) |  |  |
| 2 or more | 557 (22.8) | 132 (25.9) |  |  |
| Alcohol use, ml/week (mean±SD) | 7.08±9.73 | 7.04±10.14 | 0.01 | 0.938 |
| BMI - mean±SD | 25.54±4.77 | 25.75±4.93 | 1.31 | 0.252 |
| *Clinical characteristics* | | | | |
| Antidepressant use, yes – n(%) | 629 (25.8) | 108 (21.2) | 4.67 | 0.031 |
| Severity of MDD, IDS-SR_30_ total score | 20.01±14.01 | 19.79±11.50 | 0.14 | 0.714 |
| Comparisons based on one-way ANOVA for continuous variables and chi-square test for numeric variables. Analysis performed choosing randomly one member from the N=271 families present in the dataset.  SD, standard deviation; MET, Metabolic Equivalent of Task; BMI, Body Mass Index; MDD, Major Depressive Disorder; HC, Healthy Control; IDS-SR_30_, Inventory of Depressive Symptoms, Self Rated 30 items | | | | |

## **Metabolite profiling and data processing**

After an overnight fast, EDTA plasma samples were collected and stored in aliquots at -80°C until further analysis. Samples were sent in two batches to the USA. Metabolic profiles were measured using the untargeted metabolomics platform from Metabolon Inc (Durham, NC)^[1]^. Briefly, plasma samples were divided into four fractions; two for ultra-highperformance liquid chromatographytandem mass spectrometry (UPLC-MS/MS; positive ionization), one for UPLC-MS/MS (negative ionization), and one for a UPLC-MS/MS polar platform (negative ionization). Compounds were identified using an internal spectral database.

Peaks were quantified using the area-under-the-curve in the spectra as well as the raw metabolite data set, which was measured in 29 batches, included measures for 1367 metabolites in 5181 NESDA and 1008 reference samples (well characterized pooled human plasma samples: NIST SRM 1950 (n=288; 8-16 per batch) and Metabolon reference pooled plasma samples (n=720; 20-40 per batch)) and 1367 metabolites. NIST (National Institute of Standards and Technology) samples were used to calculate and control for technical measurement variability across the 29 batches. One experimental sample and one reference sample with high missingness (> 5SD + mean missingness) were excluded from the dataset. If outliers or apparent measurement issues within one plate or several plates within one batch were observed, all values on that plate were set to ‘NA’ to not affect subsequent batch correction. We batch corrected data by normalizing each metabolite value to the batch median of the metabolite measured in the NIST reference samples and then excluded those metabolites that still had a technical measurement variability of >30% using the Metabolon reference plasma samples. For metabolites, for which the metabolite was not detected in NIST samples, we used the overall batch median for normalization instead (affected metabolites are tagged). Next, we restricted the dataset to metabolites with missingness below 30% to ensure robust imputation results^[2]^. We then tested if missingness in any of the remaining 820 metabolites accumulated in one of the three measurement waves (baseline, 6-, or 9-year follow up) using a Fisher’s exact test. As this was not the case, we jointly imputed all waves using a k-nearest neighbor approach (k=10). Before statistical analysis, we log2 transformed the final dataset.

# **SUPPLEMENTARY RESULTS**

## **Supplementary Figure 2 (Figure S2). Distribution of metabolite levels.**

Y-axes: frequency of metabolite level in total sample.


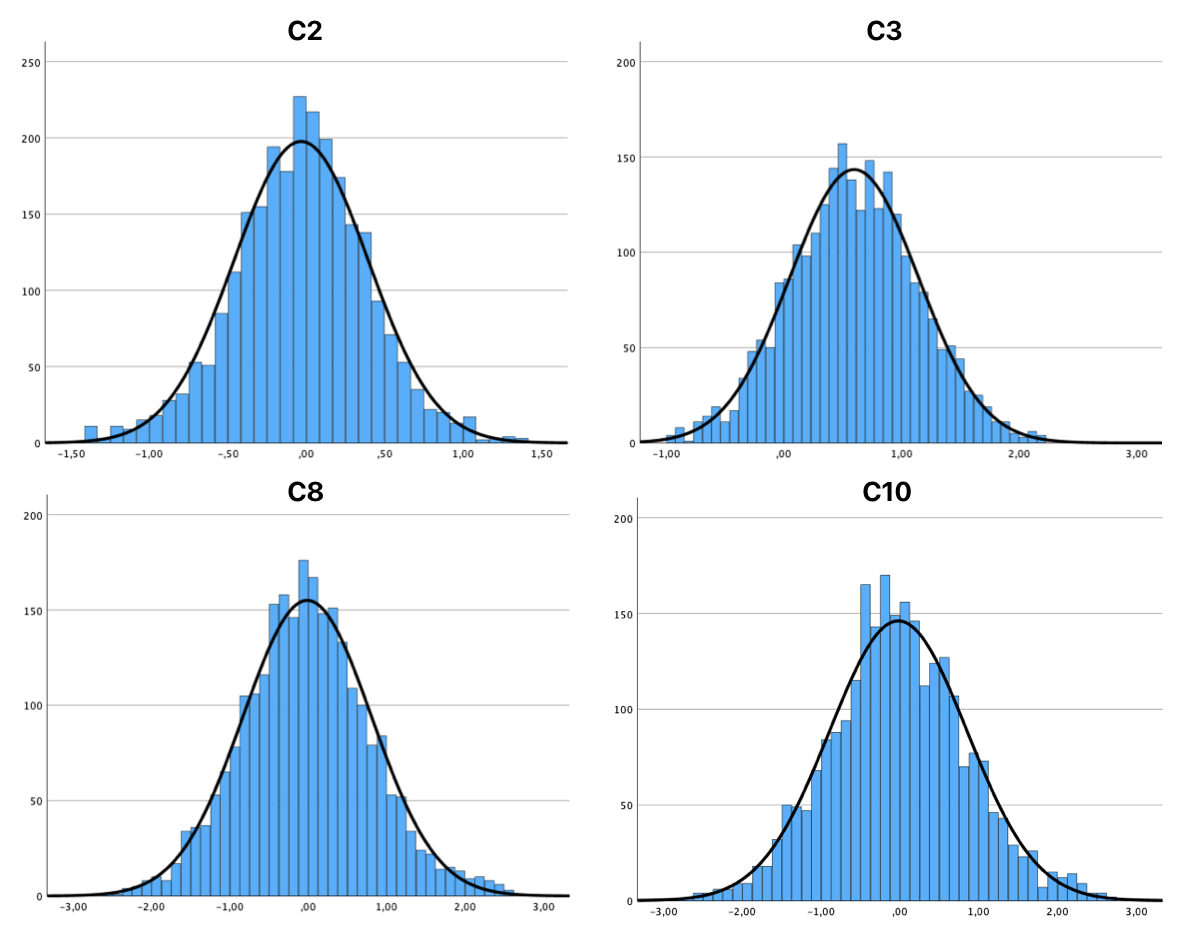


## **Supplementary Figure 3 (Figure S3). Levels of correlation of ACs metabolites.**

The heatmap shows Person’s r correlation coefficients for each pair, with colour filling underlying the level of correlation, from 1 (red) to -1 (blue). All the correlations are statistically significant, with p<0.001.

**
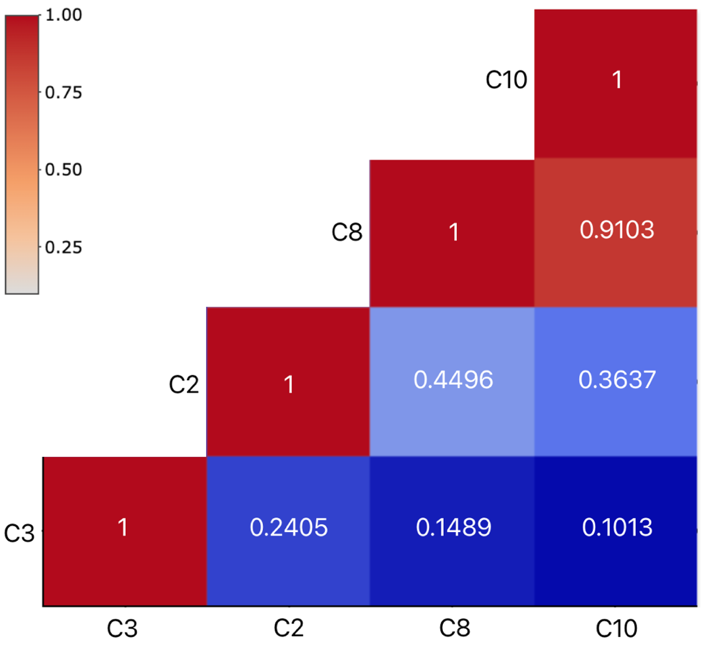
**

## **Supplementary Figure 4 (Figure S4). Levels of correlation of the three symptom scales: atypical energy-related (AES), anhedonic and melancholic.**

The heatmap shows Person’s r correlation coefficients for each pair, with colour filling underlying the level of correlation, from 1 (red) to -1 (blue). All the correlations are statistically significant.


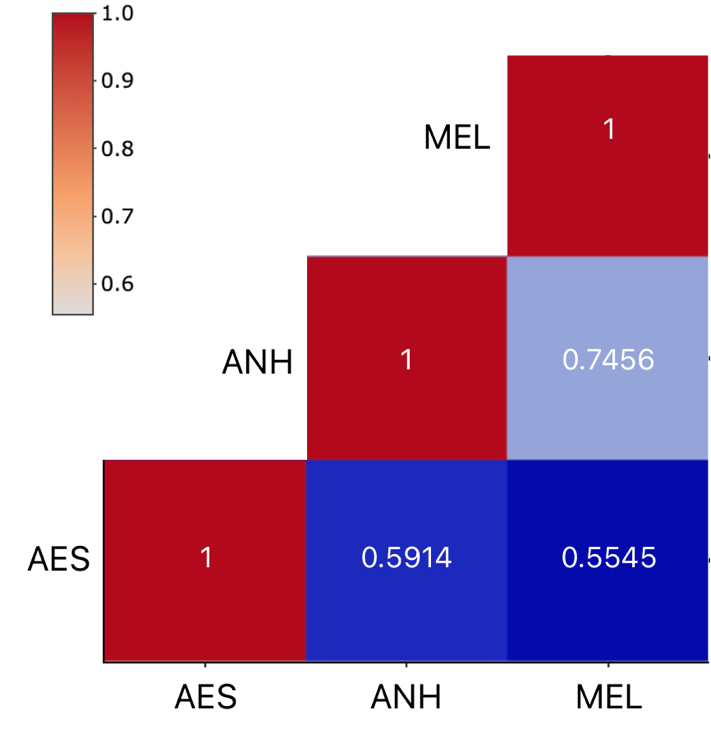


**Supplementary Figure 5 (Figure S5). Restricted cubic spline for C2 and the three symptom profiles.**

Dashed lines the fitted spline of C2 distribution across the three scales scores. Vertical lines illustrate the cutoff values that divide the scores in tertiles. AES: cut-offs -0.80 / 0.31; N I tertile 863 (34%), N II tertile 974 (38%), N III tertile 764 (30%). Anhedonic: cut-offs -0.54 / 0.28; N I tertile 1208 (47%), N II tertile 688 (27%), N III tertile 678 (26%). Melancholic: cut-offs -0.60 / 0.45; N I tertile 1057 (41%), N II tertile 760 (30%), N III tertile 757 (29%).


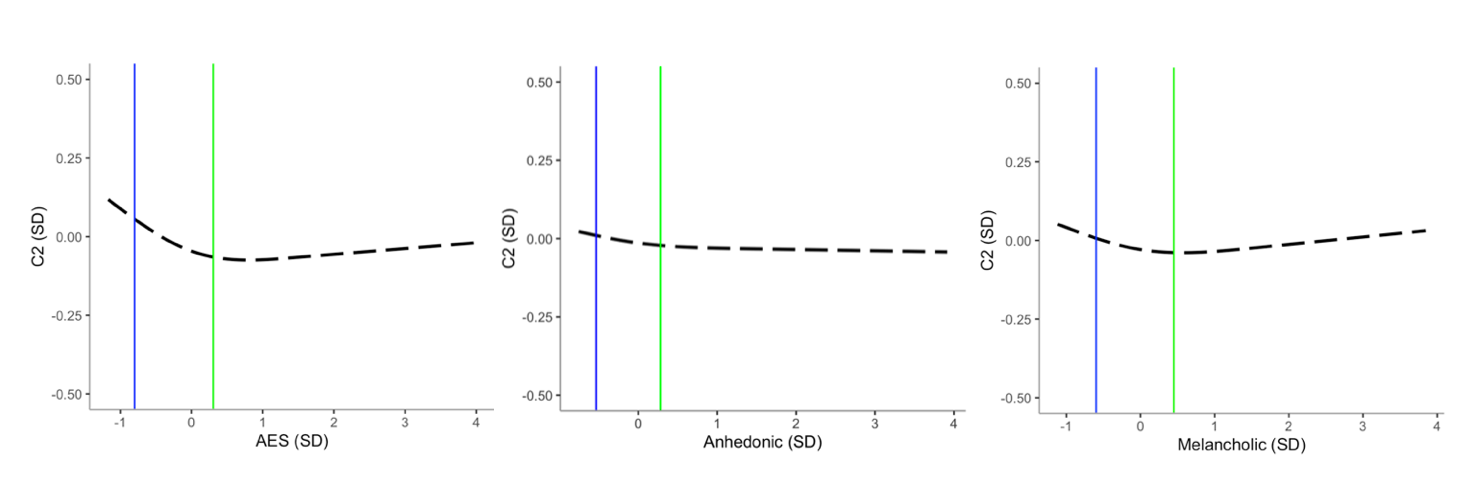


## **Supplementary Table 2. Estimated means (and SE) of C2 levels after excluding participants with antidepressant use.**

| **Table S2.** Estimated means (and SE) of C2 levels among the three diagnostic status groups after excluding participants with antidepressant use (N=1916) | | | | | | | |
| --- | --- | --- | --- | --- | --- | --- | --- |
| Metabolite (log2) | MDD current | | MDD remitted | | HC | | P |
|  | mean | SE | mean | SE | mean | SE |  |
| C2 | -0.04^A^ | 0.02 | -0.03^B^ | 0.02 | 0.04^A,B^ | 0.02 | **1.20e-3** |
| Covariates –shipment, age, sex, education. | | | | | | | |
| Pairwise comparison: groups with the same letter (A, B) for each model are significantly different (A, P=9.0e-4, d=-0.2; B, P=5.3e-3, d=-0.2) | | | | | | | |
| MDD, major depressive disorder; HC, healthy control; P, level of significance; SE, standard error. | | | | | | | |

## **Supplementary Table 3. Association of C3 levels (divided in tertiles) with symptom profile.**

| **Table S3.** Association of C2 levels (divided in tertiles) with symptom profile.  AES: cut-offs -0.80 / 0.31; N I tertile 863 (34%), N II tertile 974 (38%), N III tertile 764 (30%).  Anhedonic: cut-offs -0.54 / 0.28; N I tertile 1208 (47%), N II tertile 688 (27%), N III tertile 678 (26%).  Melancholic: cut-offs -0.60 / 0.45; N I tertile 1057 (41%), N II tertile 760 (30%), N III tertile 757 (29%). | | | | | |
| --- | --- | --- | --- | --- | --- |
| Metabolite | Symptom profile | Tertiles | Estimate | SE | P |
| C2 | AES | I | -0.01 | 0.05 | **2.18e-2** |
|  |  | II | -0.01 | 0.05 | **3.28e-2** |
|  | Anhedonic | I | 0.01 | 0.05 | 8.55e-1 |
|  |  | II | -0.03 | 0.05 | 5.99e-1 |
|  | Melancholic | I | -0.05 | 0.05 | 3.21e-1 |
|  |  | II | -0.07 | 0.05 | 1.55e-1 |
| Covariates – shipment, age, sex, education. SE, Standard error; P, level of significance; AES, atypical energy-related symptoms | | | | | |

##

## **Supplementary Table 4. Comparison of baseline characteristics of the subjects included at 6 year and those lost to follow-up from the main NESDA sample.**

| **Table S4**: Comparison of baseline characteristics of the subjects included at 6 year and those lost to follow-up from the main NESDA sample. | | | | |
| --- | --- | --- | --- | --- |
|  | *Present at 6y (1567)* | *Not present at 6y (796)* | *F / X2* | *P* |
| *Sociodemographic characteristics* | | | | |
| Age, years - mean±SD | 42.57±12.85 | 41.01±12.99 | 7.58 | 0.006 |
| Gender - n(%) |  |  | 0.01 | 0.930 |
| Male | 542 (34.6) | 350 (34.8) |  |  |
| Female | 1025 (65.4) | 657 (65.2) |  |  |
| Level of education, years - mean±SD | 12.57±3.25 | 11.62±3.22 | 45.73 | <0.001 |
| *Lifestyle and health* | | | | |
| Smoking status - n(%) |  |  | 9.66 | 0.002 |
| No smoker | 1033 (65.9) | 603 (59.9) |  |  |
| Current smoker | 534 (34.1) | 404 (40.1) |  |  |
| Physical activity - mean±SD |  |  |  |  |
| MET total | 3833.80  ±3169.20 | 3473.31 ±2789.51 | 8.03 | 0.005 |
| Chronic diseases - n(%) |  |  | 14.8 | <0.001 |
| None | 735 (46.9) | 439 (43.6) |  |  |
| One disease | 518 (33.1) | 301 (29.9) |  |  |
| 2 or more | 314 (20.0) | 267 (26.5) |  |  |
| Alcohol use, ml/week (mean±SD) | 7.19±9.60 | 6.74±10.10 | 1.08 | 0.300 |
| BMI - mean±SD | 25.41±4.78 | 25.78±4.81 | 3.13 | 0.077 |
| *Clinical characteristics* | | | | |
| Antidepressant use, yes – n(%) | 398 (25.4) | 260 (25.8) | 0.06 | 0.811 |
| Severity of MDD, IDS-SR_30_ total score | 18.90±13.49 | 24.51±14.35 | 83.83 | <0.001 |
| Comparisons based on one-way ANOVA for continuous variables and chi-square test for numeric variables. Analyses included only subjects from the main NESDA sample with potentially available at multiple time points. The sample of siblings were excluded from the analysis because assessed only at a unique time point during the 9-year follow-up.  SD, standard deviation; MET, Metabolic Equivalent of Task; BMI, Body Mass Index; MDD, Major Depressive Disorder; HC, Healthy Control; IDS-SR_30_, Inventory of Depressive Symptoms, Self Rated 30 items | | | | |

## **Supplementary Table 5. Association of metabolite levels with MDD severity and symptom profiles for pooled data.**

| **Table S5.** Association of metabolites levels with MDD severity and symptom profiles. Pooled data, N=4141 | | | | | | | | | | | |
| --- | --- | --- | --- | --- | --- | --- | --- | --- | --- | --- | --- |
| Metabolite |  | | Main model | | | Derived model^a^:  baseline estimates | | | Derived model^a^:  6-year follow-up estimates | | |
|  |  |  | β | SE | P | β | SE | P | β | SE | P |
| C2 | Diagnostic status | Current MDD | -0.07 | 0.02 | **1.03e-4** | -0.09 | 0.02 | **2.88e-6** | -0.011 | 0.03 | 7.00e-1 |
|  |  | Remitted MDD | -0.04 | 0.02 | **1.06e-2** | -0.09 | 0.02 | **2.14e-5** | 0.02 | 0.02 | 4.82e-1 |
|  | MDD severity | IDS-SR_30_ total | -0.001 | 0.001 | 1.86e-1 | -0.002 | 0.001 | **9.39e-3** | 0.001 | 0.001 | 1.06e-1 |
|  | Symptom profile | AES | -0.01 | 0.003 | **1.99^e^-2** | -0.01 | 0.003 | **1.29e-2** | 0.002 | 0.004 | 5.82e-1 |
|  |  | Anhedonic | -0.002 | 0.004 | 6.60^e^-1 | -0.01 | 0.004 | 1.74e-1 | 0.01 | 0.01 | 1.26e-1 |
| C3 | Diagnostic status | Current MDD | 0.03 | 0.02 | 2.07e-1 | 0.03 | 0.02 | 1.79e-1 | 0.01 | 0.03 | 7.21e-1 |
|  |  | Remitted MDD | 0.04 | 0.02 | 5.93e-2 | 0.02 | 0.03 | 5.57e-1 | 0.07 | 0.03 | **9.54e-3** |
|  | MDD severity | IDS-SR_30_ total | 0.002 | 0.001 | **1.24e-2** | 0.002 | 0.001 | **2.52e-2** | 0.002 | 0.001 | 6.77e-2 |
|  | Symptom profile | AES | 0.01 | 0.003 | **1.34e-3** | 0.01 | 0.003 | **2.32e-3** | 0.01 | 0.01 | 6.88e-2 |
|  |  | Anhedonic | 0.01 | 0.004 | 5.62e-2 | 0.01 | 0.01 | **2.87e-2** | 0.002 | 0.01 | 7.33e-1 |
| Covariates – shipment, age, sex, education. | | | | | | | | | | | |
| n, number of observations; SE, Standard error; P, level of significance; AES, atypical energy-related symptoms. | | | | | | | | | | | |
| ^a^Derived model: estimates derived by modelling depression-by-wave interactions | | | | | | | | | | | |

## **Supplementary Table 6. STROBE Statement.**

| Table S6. STROBE statement. | | | |
| --- | --- | --- | --- |
|  | Item No. | Recommendation | Page  No. |
| **Title and abstract** | 1 | (*a*) Indicate the study’s design with a commonly used term in the title or the abstract | 1 |
|  |  | (*b*) Provide in the abstract an informative and balanced summary of what was done and what was found | 2 |
| Introduction | | | |
| Background/rationale | 2 | Explain the scientific background and rationale for the investigation being reported | 3/4 |
| Objectives | 3 | State specific objectives, including any prespecified hypotheses | 5 |
| Methods | | | |
| Study design | 4 | Present key elements of study design early in the paper | 6 |
| Setting | 5 | Describe the setting, locations, and relevant dates, including periods of recruitment, exposure, follow-up, and data collection | 6 |
| Participants | 6 | *Cohort study*—Give the eligibility criteria, and the sources and methods of selection of participants. Describe methods of follow-up  *Case-control study*—Give the eligibility criteria, and the sources and methods of case ascertainment and control selection. Give the rationale for the choice of cases and controls  *Cross-sectional study*—Give the eligibility criteria, and the sources and methods of selection of participants | 6 |
| Variables | 7 | Clearly define all outcomes, exposures, predictors, potential confounders, and effect modifiers. Give diagnostic criteria, if applicable | 6-8 |
| Data sources/ measurement | 8* | For each variable of interest, give sources of data and details of methods of assessment (measurement). Describe comparability of assessment methods if there is more than one group | 6-8 and suppl. |
| Bias | 9 | Describe any efforts to address potential sources of bias | 6-8 |
| Study size | 10 | Explain how the study size was arrived at | 6 |
| Quantitative variables | 11 | Explain how quantitative variables were handled in the analyses. If applicable, describe which groupings were chosen and why | 6/7 |
| Statistical methods | 12 | (*a*) Describe all statistical methods, including those used to control for confounding | 9-10 |
|  |  | (*b*) Describe any methods used to examine subgroups and interactions |  |
|  |  | (*c*) Explain how missing data were addressed |  |
|  |  | (*d*) *Cohort study*—If applicable, explain how loss to follow-up was addressed  *Case-control study*—If applicable, explain how matching of cases and controls was addressed  *Cross-sectional study*—If applicable, describe analytical methods taking account of sampling strategy |  |
|  |  | (*e*) Describe any sensitivity analyses |  |
| **Results** | | | |
| Participants | 13* | (a) Report numbers of individuals at each stage of study—eg numbers potentially eligible, examined for eligibility, confirmed eligible, included in the study, completing follow-up, and analysed | 11 |
|  |  | (b) Give reasons for non-participation at each stage |  |
|  |  | (c) Consider use of a flow diagram |  |
| Descriptive data | 14* | (a) Give characteristics of study participants (eg demographic, clinical, social) and information on exposures and potential confounders | 11  (+table1) |
|  |  | (b) Indicate number of participants with missing data for each variable of interest |  |
|  |  | (c) *Cohort study*—Summarise follow-up time (eg, average and total amount) |  |
| Outcome data | 15* | *Cohort study*—Report numbers of outcome events or summary measures over time | 11 – 13  (+table2) |
|  |  | *Case-control study—*Report numbers in each exposure category, or summary measures of exposure |  |
|  |  | *Cross-sectional study—*Report numbers of outcome events or summary measures |  |
| Main results | 16 | (*a*) Give unadjusted estimates and, if applicable, confounder-adjusted estimates and their precision (eg, 95% confidence interval). Make clear which confounders were adjusted for and why they were included | 11– 13  (+table2) |
|  |  | (*b*) Report category boundaries when continuous variables were categorized |  |
|  |  | (*c*) If relevant, consider translating estimates of relative risk into absolute risk for a meaningful time period |  |
| Other analyses | 17 | Report other analyses done—eg analyses of subgroups and interactions, and sensitivity analyses | 14 |
| **Discussion** | | | |
| Key results | 18 | Summarise key results with reference to study objectives | 17 |
| Limitations | 19 | Discuss limitations of the study, taking into account sources of potential bias or imprecision. Discuss both direction and magnitude of any potential bias | 21 |
| Interpretation | 20 | Give a cautious overall interpretation of results considering objectives, limitations, multiplicity of analyses, results from similar studies, and other relevant evidence | 17 - 20 |
| Generalisability | 21 | Discuss the generalisability (external validity) of the study results | 21 |
| **Other information** | | | |
| Funding | 22 | Give the source of funding and the role of the funders for the present study and, if applicable, for the original study on which the present article is based |  |

# **REFERENCES**

1. Evans, Anne, Mitchell, M., Bridgewater, B., Liu, Q., Stewart, S., Dai, H., Dehaven, C., & Miller, L. (2014). High Resolution Mass Spectrometry Improves Data Quantity and Quality as Compared to Unit Mass Resolution Mass Spectrometry in High-Throughput Profiling Metabolomics. *Metabolomics*, *4*.

2. Do, K. T., Wahl, S., Raffler, J., Molnos, S., Laimighofer, M., Adamski, J., Suhre, K., Strauch, K., Peters, A., Gieger, C., Langenberg, C., Stewart, I. D., Theis, F. J., Grallert, H., Kastenmüller, G., & Krumsiek, J. (2018). Characterization of missing values in untargeted MS-based metabolomics data and evaluation of missing data handling strategies. *Metabolomics: Official Journal of the Metabolomic Society*, *14*(10), 128. https://doi.org/10.1007/s11306-018-1420-2

3. Dambrova, M., Makrecka-Kuka, M., Kuka, J., Vilskersts, R., Nordberg, D., Attwood, M. M., Smesny, S., Sen, Z. D., Guo, A. C., Oler, E., Tian, S., Zheng, J., Wishart, D. S., Liepinsh, E., & Schiöth, H. B. (2022). Acylcarnitines: Nomenclature, Biomarkers, Therapeutic Potential, Drug Targets, and Clinical Trials. *Pharmacological Reviews*, *74*(3), 506–551. https://doi.org/10.1124/pharmrev.121.000408

4. Li, S., Gao, D., & Jiang, Y. (2019). Function, detection and alteration of acylcarnitine metabolism in hepatocellular carcinoma. In *Metabolites* (Vol. 9, Fascicolo 2). https://doi.org/10.3390/metabo9020036
